# Should proton pump inhibitors be used for glucocorticoid-related upper gastrointestinal bleeding prevention in patients with autoimmune liver disease? A real-world cohort study

**DOI:** 10.3389/fimmu.2026.1878948

**Published:** 2026-09-10

**Authors:** Rui Zhang, Lina Cui, Dawei Ding, Guanya Guo, Changcun Guo, Gui Jia, Yulong Shang, Ying Han

**Affiliations:** State Key Laboratory of Holistic Integrative Management of Gastrointestinal Cancers, National Clinical Research Center for Digestive Diseases, Xijing Hospital of Digestive Diseases, The Air Force Military Medical University, Xi’an, Shaanxi, China

**Keywords:** autoimmune hepatitis, autoimmune liver disease, glucocorticoid, proton pump inhibitors, risk factors, upper gastrointestinal bleeding

## Abstract

**Background and aims:**

Glucocorticoids (GCs) as a key therapeutic option for autoimmune liver diseases (AILDs) are associated with an increased risk of upper gastrointestinal bleeding (UGIB). Proton pump inhibitors (PPIs) are widely used for the prevention and treatment of drug-related UGIB. However, it is unclear whether PPI can be used for the prevention of GC-related UGIB in patients with AILD. The aim of the study was to identify risk factors for UGIB and evaluate the prophylactic efficacy of PPI in patients with AILD undergoing GC.

**Methods:**

We retrospectively compared the occurrence of UGIB and PPI use in patients with AILD receiving GC between January 2005 and May 2025. Univariate and multivariate Cox regression were analyzed to identify independent influencing factors. Propensity score matching (PSM) was utilized to evaluate the prophylactic effect of PPI administration at different time points.

**Results:**

A total of 364 patients with AILD treated with GC were included with a median follow-up duration of 29 months. Thirty-two patients (8.8%) experienced UGIB. Compared to patients without UGIB, the UGIB group had a higher proportion of PBC+AIH (90.6% vs. 64.8%; *p* = 0.001) and moderate to severe esophagogastric varices (56.3% vs. 19.9%; *p* < 0.001), as well as higher alkaline phosphatase (ALP) (269 vs. 153; *p* < 0.001) and immunoglobulin M (IgM) (3.82 vs. 2.28; *p* = 0.007) and lower platelet count (PLT) (100 vs. 121; *p* = 0.015). After multivariate Cox adjustment, concurrent with PLT (HR = 0.993, 95% CI: 0.986–1.000, *p* = 0.048), moderate to severe esophagogastric varices (HR = 3.876, 95% CI: 1.798–8.355, *p* = 0.001) were independently associated with UGIB. In this cohort, PPI users had a higher risk of UGIB; however, this association may be driven by confounding factors (log-rank *p* = 0.008). The duration of PPI use did not significantly affect the cumulative risk of UGIB (log-rank *p* = 0.426). Furthermore, neither baseline (log-rank *p* = 0.343), within 1 year after baseline (log-rank *p* = 0.132), nor PPI use for more than 1 year after baseline (log-rank *p* = 0.195) significantly reduced the cumulative risk of UGIB.

**Conclusions:**

In our real-world cohort, PPI use does not prevent GC-related UGIB among patients with AILD and may even be associated with a higher bleeding risk; however, this observed association is likely driven by confounding factors and should not be interpreted as a causal relationship.

## 1 Introduction

Autoimmune liver diseases (AILDs), primarily including autoimmune hepatitis (AIH), primary biliary cholangitis (PBC), primary sclerosing cholangitis (PSC), and overlap syndrome (OS), have an increasing incidence in most parts of the world (1). Glucocorticoids (GCs) are recommended as a first-line or second-line induction therapy and are a key therapeutic option for AILD (2–4).

However, long-term GC therapy is associated with numerous adverse effects and complications, including upper gastrointestinal bleeding (UGIB), diabetes, osteoporosis, and infections (5). A large population-based study evaluating the risks of short-term oral steroid bursts (≤14 days), which included 15,859,129 patients, found that individuals taking steroids had a higher incidence of UGIB compared to non-users, with the peak incidence occurring within the first month of initiating steroid therapy (6). A Norwegian meta-analysis encompassing 159 studies and 804 patients also indicated that GC use increases the risk of gastrointestinal bleeding. The analysis revealed that among hospitalized patients, GC users had a 40% increased odds ratio (OR 1.43, 95% CI 1.22–1.66) for gastrointestinal bleeding or perforation compared to placebo users (7). Additionally, GC treatment elevates the risk of peptic ulcer bleeding. A nationwide cohort study of 8,894 patients aged ≥20 years with newly diagnosed peptic ulcers showed that those taking higher doses of GC had a greater risk of peptic ulcer bleeding than those on lower doses, and patients combining GC with non-steroidal anti-inflammatory drugs (NSAIDs)/aspirin faced a higher risk than those using GC alone (8).

Proton pump inhibitors (PPIs) are widely used for the prevention and treatment of drug-related UGIB. Evidence supports their efficacy in patients taking NSAIDs, antiplatelet agents, or anticoagulants (9–11). However, there is currently no evidence to confirm that PPI has a preventive effect on UGIB related to GC therapy.

In addition, in patients with advanced liver disease, PPI use has been linked to an increased risk of complications such as spontaneous bacterial peritonitis, hepatic encephalopathy, and progression to acute-on-chronic liver failure (12–14). This indicates that taking PPI can seriously affect the prognosis of patients with AILD who are prone to developing chronic liver disease.

Therefore, whether taking PPI can reduce the risk of GC-related UGIB and bring benefits to patients with AILD still needs further exploration. For this reason, we conducted this retrospective study to investigate the influencing factors for UGIB and assess the prophylactic efficacy of PPI in patients with AILD receiving GC therapy.

## 2 Materials and methods

### 2.1 Study population

This study retrospectively analyzed a cohort of patients with AILD who commenced GC therapy at Xijing Hospital between January 2005 and May 2025. The patients were initially identified by querying the electronic medical record and prescription databases for patients who had received at least one prescription for systemic GCs between January 2005 and May 2025, with a concurrent diagnosis of AILD. In our cohort, patients are followed up every 3–6 months with laboratory tests and detailed symptom interviews during visits, in order to confirm as thoroughly as possible whether they have had gastrointestinal bleeding.

Participants were included if they (i) fulfilled the latest diagnostic criteria for AIH, PBC, or OS established by the Chinese Medical Association’s Hepatology Society and the American Association for the Study of Liver Diseases; (ii) received a GC treatment (including prednisone, methylprednisolone, or hydrocortisone); and (iii) were above 18 years of age. Exclusion criteria were as follows: (i) GC treatment duration <30 days (to investigate whether PPIs should be used in patients with AILD who are on long-term GC therapy); (2) incomplete medication records; (3) missing baseline data; and (4) loss to follow-up. After screening, 364 patients were ultimately enrolled.

### 2.2 Study design

A single-center retrospective study was conducted. Firstly, we compared the clinical characteristics of the first administration of GC between with UGIB and without UGIB, and analyzed the risk factors for UGIB in patients with AILD treated with GC. Secondly, we evaluated the preventive effect of PPI on UGIB from three aspects: the applicability of administration, the duration of treatment, and the timing of addition.

The baseline clinical characteristics and laboratory test values were collected from the electronic medical records at their first receiving GC. The clinical data collected comprised baseline demographics, comorbidities, key laboratory tests (including liver function and coagulation parameters), abdominal ultrasound and gastroduodenoscopy findings, carbon-13 urea breath test results, and the dosage and duration of GC and PPI therapy.

When evaluating the impact of the PPI administration time point on UGIB, we categorized patients taking PPI into three groups based on the timing of PPI initiation relative to GC start: at baseline (PPI started concurrently with GC), within 1 year after baseline (PPI started between 1 week and 1 year after baseline), or beyond 1 year after baseline (PPI added more than 1 year after baseline). The Non-PPI group data were collected at baseline, 5 months after baseline, and 30 months after baseline, corresponding to the three groups, respectively. Using the statistically significant factors identified from the univariate Cox analysis as matching covariates. A 1:2 propensity score matching (PSM) with a caliper width of 0.3 of the standard deviation of the logit of the propensity score was performed between patients taking PPI and those who did not.

The primary outcome was the occurrence of UGIB during GC therapy. UGIB was defined as meeting both of the following criteria: (1) a documented clinical presentation of hematemesis (either bright red or coffee-ground) and/or melena in the electronic medical records, and (2) a confirmed drop in hemoglobin level of >2 g/dL within 48 hours of symptom onset compared to a recent baseline. The follow-up period started from the initiation of GC therapy (baseline) and continued until the first occurrence of any of the following events: the primary outcome, death from any cause, or the study cutoff date.

The assessment of esophageal and gastric variceal severity refers to the classification method for the severity of esophageal and gastric variceal bleeding recommended in the 2023 Chinese Association of Hepatology Guidelines for the Prevention and Treatment of Esophageal and Gastric Variceal Bleeding due to Liver Cirrhosis and Portal Hypertension (15). It is classified into three levels: mild, moderate, and severe. This study focused on the impact of moderate-to-severe esophagogastric varices on UGIB in patients with AILD receiving GC therapy.

### 2.3 Statistical analysis

Continuous variables were presented as medians with interquartile ranges and compared using the Mann–Whitney *U* test. Categorical variables were expressed as numbers (percentages) and compared using the Chi-square or Fisher’s exact test. Univariate and multivariate Cox proportional hazards models were used to identify independent risk factors for UGIB. Variables with *p* < 0.05 in the univariate analysis were included in the multivariate model. The cumulative incidence of UGIB was compared using Kaplan–Meier curves with the log-rank test. A two-sided *p*-value of <0.05 was considered statistically significant. All analyses were performed using SPSS 25.0 and R software (version 4.5.1).

Missing data were handled according to the following principles: (1) Baseline variables missing: At enrollment, if key baseline variables were missing for a patient, the patient was considered a screening failure and was not included in this analysis. (2) Intermediate follow-up variables missing: A follow-up visit window of ±4 weeks was applied. If data were available within the window, they were recorded using the actual date; if data were completely missing within the window, no imputation was performed, and the data were treated as “missing” at that time point, with only the patient’s data from other follow-up visits being included in the analysis. (3) Primary outcome variables missing: If the primary outcome had not occurred by the study cutoff date and the patient was known to be alive, the patient was defined as censored, with the censoring date set as the date of the last known follow-up.

To assess the impact of missing data on the robustness of the results, we performed sensitivity analyses. The primary analysis of the outcome was based on the missing-at-random assumption. In the sensitivity analysis, we applied a worst-case imputation approach, assuming that all censored patients had experienced the outcome event, to test the stability of our conclusions. For the post-matching cohort, we compared the results prior to and after matching, and further compared the matching outcomes across different covariate adjustment sets.

## 3 Results

### 3.1 Risk factors associated with UGIB in patients with AILD receiving GC

**Figure 1:**
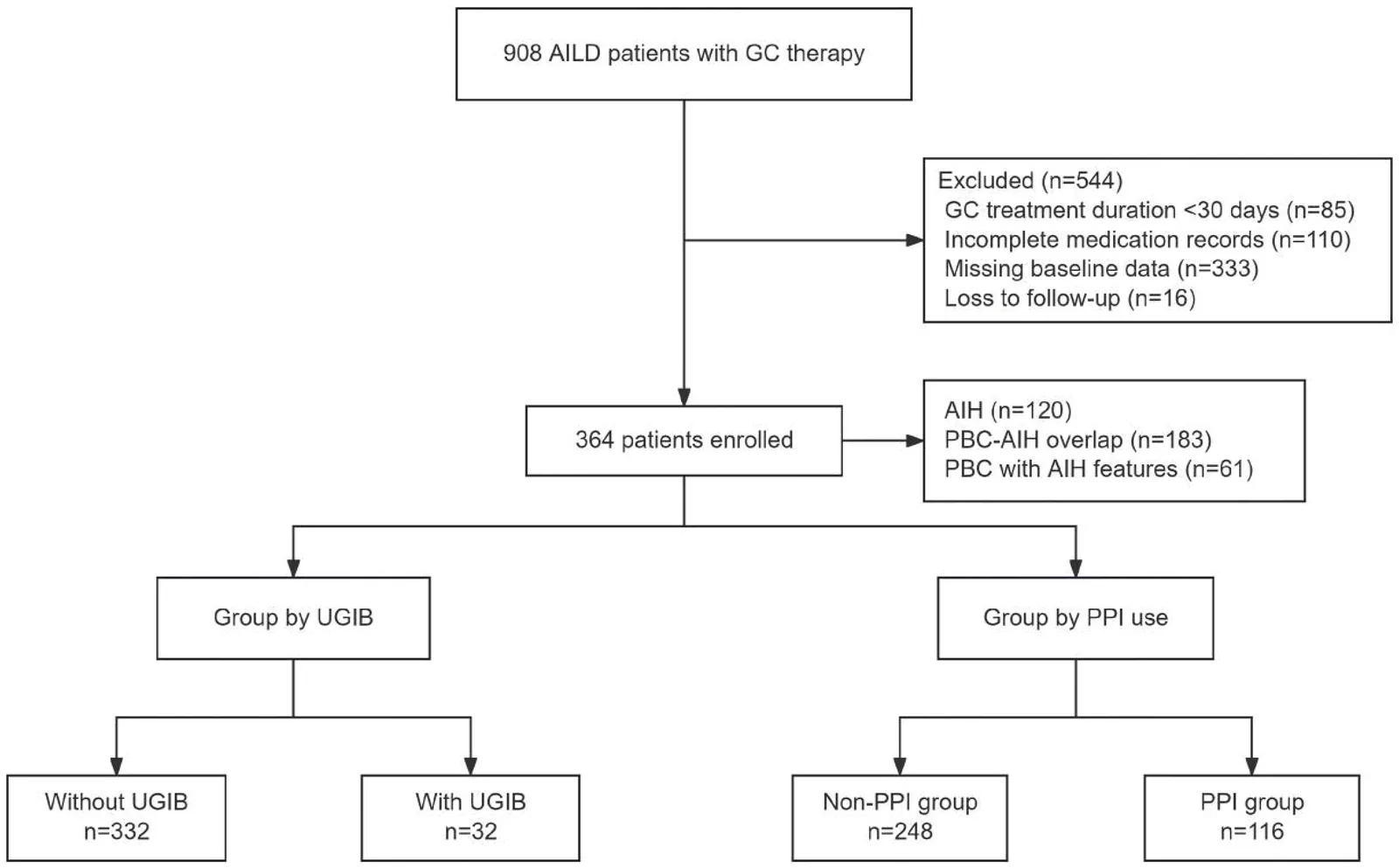
Flowchart of the study. AILD, autoimmune liver disease; AIH, autoimmune hepatitis; PBC, primary biliary cholangitis; UGIB, upper gastrointestinal bleeding; GC, glucocorticoid; PPI, proton pump inhibitor.

A total of 908 patients with AILD treated with GC were initially screened. From these, 364 patients were finally included in the study (**Figure 1** presents the flowchart of the study). For the 364 patients, 52 were censored due to missing primary outcome data before the study cutoff date, yielding a censoring rate of 14.2%. The cohort was predominantly female (89%), with a median age of 53 years and a median follow-up duration of 29 months. A total of 120 cases (33.0%) were AIH, 183 cases (50.3%) were PBC-AIH overlap, and 61 cases (16.7%) were PBC with AIH features. Both PBC-AIH overlap and PBC with AIH features were grouped together as PBC+AIH for subsequent analyses.

Among the 364 enrolled patients, 32 patients (8.8%) experienced UGIB. Compared to patients without UGIB, the UGIB group had a higher proportion of PBC+AIH (90.6% vs. 64.8%; *p* = 0.001) and moderate to severe esophagogastric varices (56.3% vs. 19.9%; *p* < 0.001). In addition, the UGIB group showed significantly higher levels of alkaline phosphatase (ALP) (269 vs. 153; *p* < 0.001) and immunoglobulin M (IgM) (3.82 vs. 2.28; *p* = 0.007) and significantly lower platelet count (PLT) (100 vs. 121; *p* = 0.015). There were no significant differences in sex, age, follow-up time, alanine aminotransferase (ALT), albumin (ALB), activated partial thromboplastin time (APTT), immunoglobulin G (IgG), C13 positivity, diabetes, hypertension, peptic ulcer, and other drug-related factors in the two groups (**Table 1**).

**Table 1:** Baseline characteristics of patients with and without UGIB.

Parameter	Total *n* = 364	Without UGIB *n* = 332	With UGIB *n* = 32	P-value
Demographic				
Female (*n*, %)	324 (89)	295 (88.9)	29 (90.6)	0.992
Age (years)	53 (47–59)	53 (47–59)	53 (49–57)	0.664
FU time (Mo)	29 (9–57)	29 (9–59)	31 (9–51)	0.625
Diagnosis (*n*, %)				0.003
AIH	120 (33.0)	117 (35.2)	3 (9.4)	
PBC+AIH	244 (67.0)	215 (64.8)	29 (90.6)	
Laboratory				
PLT (×10^9^/L)	115 (75–179)	121 (76–186)	100 (60–121)	0.015
ALT (IU/L)	60 (32–109)	59 (31–109)	76 (47–120)	0.301
ALP (IU/L)	158 (108–254)	153 (107–242)	269 (160–420)	<0.001
TBIL (μmol/L)	23.90 (14.22–48.67)	23.75 (14.10–44.55)	30.65 (16.12–66.92)	0.163
ALB (g/L)	36.15 ± 5.24	36.10 ± 5.28	36.71 ± 4.83	0.505
APTT (s)	38.45 (33.10–43.27)	38.45 (33.10–43.37)	38.40 (33.45–41.65)	0.860
INR	1.07 (0.98–1.23)	1.07 (0.99–1.23)	0.99 (0.91–1.10)	0.007
IgG (g/L)	21.20 (17.12–25.80)	21.20 (17.10–25.60)	21.60 (17.35–27.50)	0.832
IgM (g/L)	2.39 (1.41–2.30)	2.28 (1.38–3.95)	3.82 (2.05–5.67)	0.007
Complications				
C13 positivity (*n*, %)	68 (18.7)	59 (17.8)	9 (28.1)	0.151
Diabetes (*n*, %)	19 (5.2)	17 (5.1)	2 (6.3)	0.784
Hypertension (*n*, %)	38 (10.4)	35 (10.5)	3 (9.4)	0.837
Peptic ulcer (*n*, %)	18 (4.9)	15 (4.5)	3 (9.4)	0.433
MS EGV (*n*, %)	84 (23.1)	66 (19.9)	18 (56.3)	<0.001
Medication				
Initial GC dose (mg)	40 (30–40)	40 (30–40)	40 (30–40)	0.183
Duration of GC (Mo)	10 (4–25)	10 (4–25)	11 (4–22)	0.781
PPI use (*n*, %)	68 (18.7)	60 (18.1)	8 (25.0)	0.337

FU time, follow-up time; Mo, months; PLT, platelet count; ALT, alanine aminotransferase; ALP, alkaline phosphatase; TBIL, total bilirubin; ALB, albumin; APTT, activated partial thromboplastin time; INR, International Normalized Ratio; IgG, immunoglobulin G; IgM, immunoglobulin M; C13, Carbon-13 urea breath test; MS EGV, moderate to severe esophagogastric varices; GC, glucocorticoid; PPI, proton pump inhibitor.

*p*-values were between without UGIB and with UGIB.

Univariate Cox regression identified PLT (HR = 0.990, 95% CI: 0.983–0.996, *p* = 0.002), ALP (HR = 1.001, 95% CI: 1.000–1.002, *p* = 0.033), IgM (HR = 1.074, 95% CI: 1.002–1.152, *p* = 0.045), moderate to severe esophagogastric varices (HR = 6.748, 95% CI: 3.336–13.650, *p* < 0.001), and disease type as significant predictors of UGIB risk. In pairwise comparisons with AIH (HR = 5.193, 95% CI: 1.582–17.050, *p* = 0.007) as the reference group, patients with PBC+AIH had a significantly higher risk. These factors were entered into a multivariate Cox model. After adjustment, only two remained independent predictors: PLT (HR = 0.993, 95% CI: 0.986–1.000, *p* = 0.048) and moderate to severe esophagogastric varices (HR = 3.876, 95% CI: 1.798–8.355, *p* = 0.001) (**Table 2**).

**Table 2:** Factors associated with UGIB in patients with AILD receiving GC.

Variable	Univariate			Multivariate		
	HR	95% CI	*p*-value	HR	95% CI	*p*-value
Demographic						
Female	1.179	0.359–3.879	0.786			
Age (years)	1.020	0.986–1.056	0.249			
Laboratory						
PLT (×10^9^/L)	0.990	0.983–0.996	0.002	0.993	0.986–1.000	0.048
ALT (IU/L)	0.998	0.994–1.001	0.196			
ALP (IU/L)	1.001	1.000–1.002	0.033	1.001	1.000–1.002	0.200
APTT (s)	0.972	0.937–1.009	0.141			
IgG (g/L)	0.994	0.956–1.034	0.770			
IgM (g/L)	1.074	1.002–1.152	0.045	1.034	0.962–1.112	0.365
Diagnosis						
AIH	Ref	-	-	Ref	-	-
PBC+AIH	5.193	1.582–17.050	0.007	2.918	0.836–10.186	0.093
Complications						
C13 positivity	1.316	0.607–2.849	0.487			
Hypertension	0.660	0.201–2.168	0.493			
Diabetes	1.491	0.355–6.256	0.585			
Peptic ulcer	2.130	0.648–6.999	0.213			
MS EGV	6.748	3.336–13.650	<0.001	3.876	1.798–8.355	0.001
Medication						
Initial GC dose (mg)	1.009	0.970–1.049	0.669			
PPI Use	1.606	0.721–3.577	0.247			

PLT, platelet count; ALT, alanine aminotransferase; ALP, alkaline phosphatase; APTT, activated partial thromboplastin time; IgG, immunoglobulin G; IgM, immunoglobulin M; C13, Carbon-13 urea breath test; MS EGV, moderate to severe esophagogastric varices; GC, glucocorticoid; PPI, proton pump inhibitor.

### 3.2 Effect of PPI administration on the prevention of UGIB

The 116 patients taking PPIs in the follow-up time comprised the PPI group, whereas the 248 patients not receiving PPIs constituted the Non-PPI group. Risk factors including disease distribution, PLT, ALP, IgM, and the presence of moderate to severe esophagogastric varices at baseline for UGIB did not differ significantly between groups.

**Figure 2:**
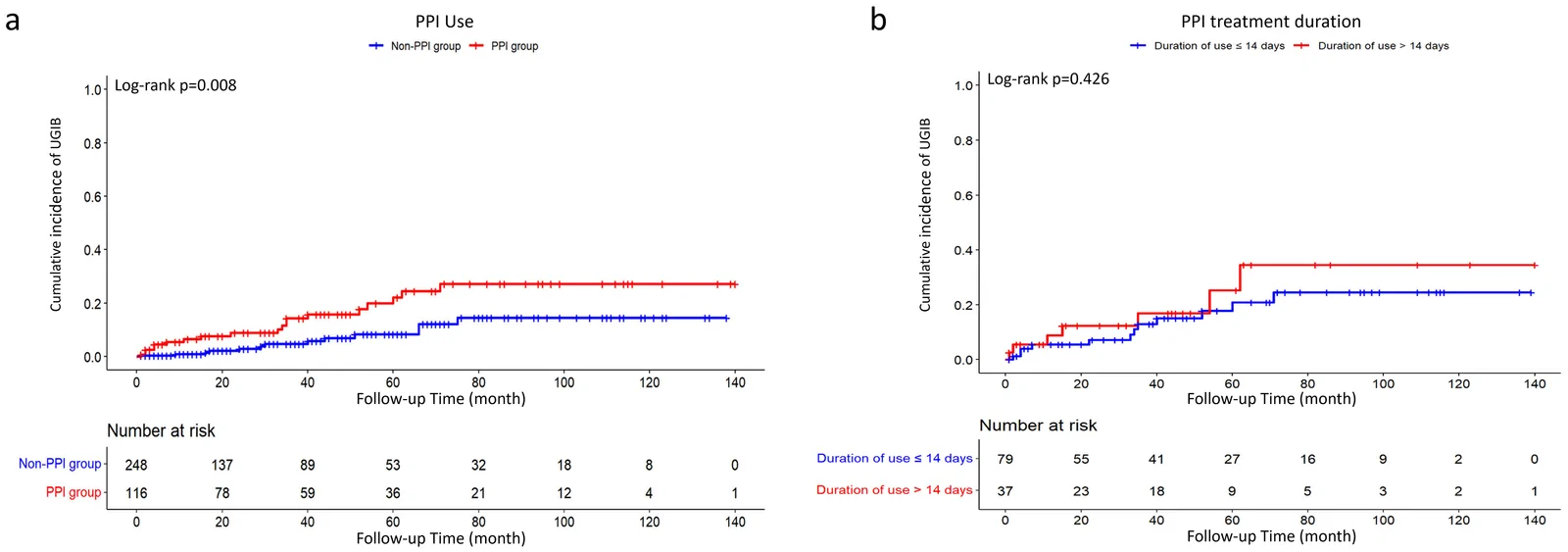
Kaplan–Meier curves for UGIB based on PPI use and treatment duration. **(a)** The comparison of patients with PPI and without PPI (log-rank *p* = 0.008). **(b)** Comparison between patients with PPI treatment duration of ≤14 days and those with PPI treatment duration of >14 days (log-rank *p* = 0.426).

Kaplan–Meier analysis revealed that the cumulative risk of UGIB was significantly higher in the PPI group (**Supplementary Table 3**, HR = 2.631, 95% CI: 1.281–5.405; **Figure 2a**, log-rank *p* = 0.008). Although this finding may be associated with disease progression, it at least indicates that PPI use cannot reduce the risk of UGIB. Moreover, the PPI group exhibited significantly higher rates of C13 positivity (53.4% vs. 2.4%; *p* < 0.001) and peptic ulcer (11.2% vs. 2.0%; *p* < 0.001) (**Supplementary Table 1**). This suggests that C13 positivity and peptic ulcer are the main reasons for PPI use in these patients. Sensitivity analyses using worst-case imputation for censored patients yielded results consistent with the primary findings (adjusted HR = 1.311, 95% CI: 0.839–2.047; log-rank *p* = 0.234), supporting the robustness of our conclusions (**Supplementary Table 4**).

### 3.3 Effect of PPI treatment duration on the prevention of UGIB

We further analyzed the impact of PPI treatment duration on UGIB in patients taking PPI. A total of 116 patients took PPI during the entire follow-up period. Patients taking PPI were divided into duration of use ≤ 14 days (*n* = 79) and duration of use > 14 days (*n* = 37). Risk factors including disease distribution, PLT, ALP, IgM, and the presence of moderate to severe esophagogastric varices at baseline for UGIB did not differ significantly between groups. The most notable difference was in C13 positivity, which was significantly more frequent in the duration of use ≤ 14 days group (67.1% vs. 24.3%, *p* < 0.001) (**Supplementary Table 2**).

After performing the Kaplan–Meier analysis, we found no significant difference in cumulative incidence of UGIB observed between those who used PPIs for ≤14 days and those who used them for >14 days. This suggests that the length of PPI treatment did not significantly influence risk of UGIB (**Supplementary Table 3**, HR = 1.212, 95% CI: 0.438–3.356; **Figure 2b**, log-rank *p* = 0.426). In sensitivity analyses, the adjusted HR for the primary outcome remained largely unchanged (HR = 2.039, 95% CI: 0.976–4.256, log-rank *p* = 0.057), supporting the robustness of our conclusions (**Supplementary Table 4**).

### 3.3 Effect of PPI administration timing on the prevention of UGIB

To control for the effect of varying PPI administration timing on its efficacy in preventing UGIB, 116 patients were divided from the PPI group into three groups based on the timing of administration: baseline (*n* = 68), within 1 year after baseline (*n* = 26), and beyond 1 year after baseline (*n* = 22). Each group was then matched with the remaining 248 from the Non-PPI group based on the statistically significant factors identified from the univariate Cox analysis in a 1:2 ratio using PSM. The PSM results for each group are presented in **Table 3**. After PSM, there were 67 complete pairs at baseline, 23 complete pairs within 1 year of baseline, and 12 complete pairs plus 5 incomplete (1:1) pairs beyond 1 year from baseline. Unmatched patients were due to either propensity score differences exceeding the prespecified caliper (0.3) with no suitable controls available, or loss to follow-up before the pseudo-baseline (beyond 1 year after baseline only).

**Table 3:** Clinical characteristics between the non-PPI group and the PPI group according to the time points of administration (after PSM).

	Non-PPI group	PPI group	*p*-value
Baseline	*n* = 134	*n* = 67	
Female (*n*, %)	113 (84.3)	58 (86.6)	0.675
Age (years)	53 (48–60)	53 (45–60)	0.803
FU time (Mo)	24 (9–55)	29 (7–54)	0.603
PBC+AIH (*n*, %)	92 (68.7)	38 (56.7)	0.095
PLT (×10^9^/L)	115 (77–163)	104 (73–167)	0.457
ALT (IU/L)	61 (31–113)	71 (37–123)	0.383
ALP (IU/L)	156 (111–256)	171 (107–303)	0.686
APTT (s)	38.80 (33.47–44.00)	40.80 (34.10–44.40)	0.548
IgG (g/L)	21.20 (16.87–27.45)	21.60 (16.70–25.90)	0.908
IgM (g/L)	2.36 (1.17–4.61)	2.32 (1.29–4.57)	0.648
MS EGV (*n*, %)	29 (21.6)	17 (25.4)	0.553
Within 1 year after baseline	*n* = 46	*n* = 23	
Female (*n*, %)	45 (97.8)	21 (91.3)	0.531
Age (years)	55 (50–59)	55 (49–59)	0.735
FU time (Mo)	19 (6–44)	38 (21–60)	0.025
PBC+AIH (*n*, %)	27 (58.7)	17 (73.9)	0.215
PLT (×10^9^/L)	125 (65–188)	138 (82–169)	0.562
ALT (IU/L)	30 (19–53)	23 (18–40)	0.333
ALP (IU/L)	109 (85–148)	120 (77–170)	0.874
APTT (s)	35.65 ± 6.18	36.82 ± 8.62	0.521
IgG (g/L)	15.85 (13.57–20.67)	17.30 (14.20–18.80)	0.441
IgM (g/L)	2.35 (1.46–3.73)	2.59 (1.65–2.95)	0.879
MS EGV (*n*, %)	12 (26.1)	6 (26.1)	1.000
Beyond 1 year after baseline	*n* = 29	*n* = 17	
Female (*n*, %)	28 (96.6)	17 (100.0)	0.630
Age (years)	52 ± 8	52 ± 8	0.832
FU time (Mo)	8 (0–42)	44 (13–90)	0.007
PBC+AIH (*n*, %)	21 (72.4)	14 (82.4)	0.686
PLT (×10^9^/L)	122 (73–192)	130 (61–204)	0.873
ALT (IU/L)	24 (15–40)	31 (18–52)	0.362
ALP (IU/L)	96 (73–191)	125 (81–172)	0.617
APTT (s)	34.30 ± 6.41	35.18 ± 6.22	0.648
IgG (g/L)	15.20 (12.10–17.05)	15.90 (14.50–18.80)	0.206
IgM (g/L)	1.92 (1.26–3.08)	1.62 (0.79–3.37)	0.767
	2.83 (1.88–4.10)	2.77 (1.95–4.33)	0.609
MS EGV (*n*, %)	4 (13.8)	4 (23.5)	0.661

FU time, follow-up time; Mo, months; PLT, platelet count; ALT, alanine aminotransferase; ALP, alkaline phosphatase; APTT, activated partial thromboplastin time; IgG, immunoglobulin G; IgM, immunoglobulin M; MS EGV, moderate to severe esophagogastric varices; PPI, proton pump inhibitors.

In the beyond 1 year after baseline group, 12 complete pairs (1:2) and 5 incomplete (1:1) pairs were formed.

**Figure 3:**
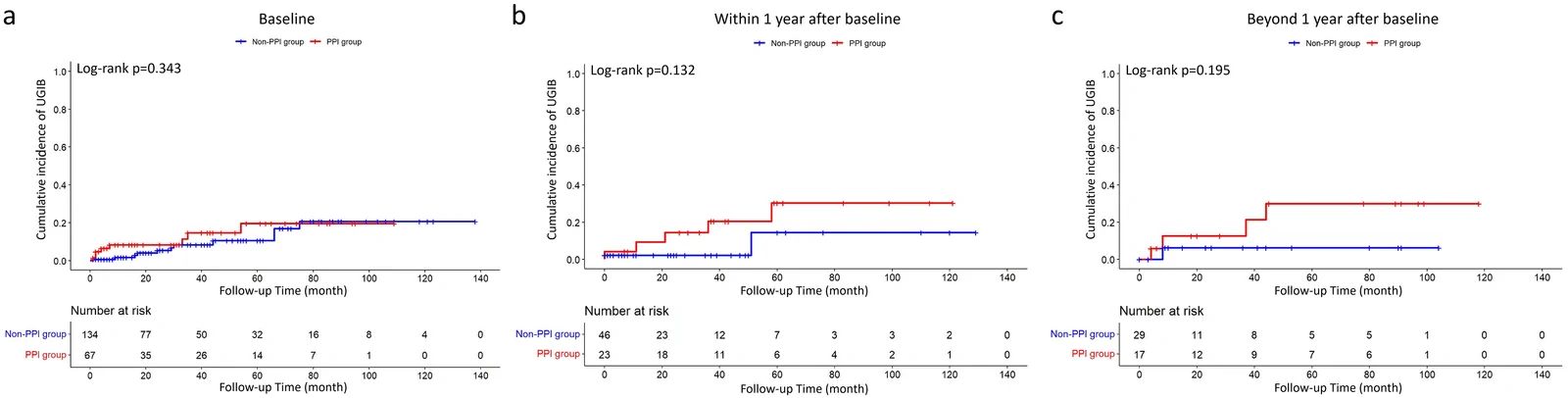
Kaplan–Meier curves for UGIB based on PPI initiation time point. **(a)** PPI use at baseline vs. Non-PPI (log-rank *p* = 0.343). **(b)** PPI initiation within 1 year after baseline vs. Non-PPI (log-rank *p* = 0.132). **(c)** PPI initiation beyond 1 year after baseline vs. Non-PPI (log-rank *p* = 0.195).

We conducted Kaplan–Meier analysis on the three groups. The analysis shows that there was no significant difference in the cumulative incidence of UGIB observed between patients who started PPIs at baseline and those who did not. These findings suggest that the addition of PPI at the baseline stage, within 1 year after the baseline, or beyond 1 year after the baseline has no significant impact on the risk of UGIB (**Supplementary Table 3**, HR = 1.539, 95% CI: 0.587–4.028; **Figure 3a**, log-rank *p* = 0.343; **Supplementary Table 3**, HR = 3.686, 95% CI: 0.633–21.470; **Figure 3b**, log-rank *p* = 0.132; **Supplementary Table 3**, HR = 3.228, 95% CI: 0.548–18.900; **Figure 3c**, log-rank *p* = 0.195). In sensitivity analyses, the adjusted HR for the primary outcome remained largely unchanged across the three time points of PPI initiation (baseline: HR = 2.002, 95% CI: 1.126–3.558, log-rank *p* = 0.018; within 1 year after the baseline: HR = 1.147, 95% CI: 0.464–2.829, log-rank *p* = 0.766; beyond 1 year after the baseline: HR = 1.197, 95% CI: 0.337–4.237, log-rank *p* = 0.780), supporting the robustness of our conclusions (**Supplementary Table 5**). To further assess the robustness of our findings to unmeasured confounding, we performed another PSM analysis re-matching with C13 and peptic ulcer included as covariates. This analysis suggests that the observed effect is relatively robust to indication confounding. Detailed results are provided in **Supplementary Table 6**.

## 4 Discussion

In our real-world cohort, we identified lower platelet count and moderate-to-severe esophagogastric varices as independent risk factors for UGIB. Neither the duration of PPI administration nor the timing of PPI initiation at various time points was associated with a reduced risk of UGIB. PPI use did not demonstrate a preventive effect against GC-related UGIB among patients with AILD, and may even be associated with a higher bleeding risk; however, this observed association is likely driven by confounding factors (such as more severe liver disease or comorbidities) and, thus, should not be interpreted as a causal relationship.

UGIB is not only a serious and life-threatening complication of liver cirrhosis but also an adverse effect in patients undergoing long-term GC therapy, accounting for a notable percentage of emergency admissions and mortality. A facility-based cross-sectional study conducted on 256 patients with cirrhosis from Sub-Saharan Africa reported that the prevalence of UGIB was 46.1%. Illness duration > 24 months, platelet count < 50,000/µL, and absence of prior endoscopy were significantly associated factors (16). Another study, which also aimed to identify predictors of the initial variceal bleeding occurrence in patients with cirrhosis, found that age over 60 years, diabetes mellitus, absence of ischemic heart disease, platelets below 130,000/μL, albumin > 2.9 g/dL, bilirubin level > 1.4 mg/dL, and Child–Pugh score B were identified as independent risk factors for the first variceal bleeding episode (17). Another study aimed at evaluating the relationship between platelet count and bleeding in patients with HBV and HCV infection also found that thrombocytopenia, hypoalbuminemia, high alkaline phosphatase level, and cirrhosis were risk factors for UGIB (18). In this study, we retrospectively analyzed 364 patients with AILD who received GC treatment to evaluate the risk factors for UGIB and the preventive effect of PPIs. We found that a lower platelet count, higher alkaline phosphatase levels, higher immunoglobulin M levels, and moderate-to-severe esophagogastric varices were significantly associated with UGIB. A lower platelet count and moderate-to-severe varices were confirmed as independent risk factors for UGIB. Although GCs are known to increase the risk of gastrointestinal bleeding (6–8, 19), neither their dosage nor treatment duration showed an independent association with bleeding in our multivariate Cox model.

Furthermore, patients with AIH combined with PBC appeared to have a higher risk of bleeding compared to those with AIH alone, which may reflect the unique pathological features of PBC, including chronic cholestasis, malabsorption of fat-soluble vitamins (particularly vitamin K), and the early development of portal hypertension. The moderate-to-severe esophagogastric varices were also strongly associated with UGIB risk, underscoring that variceal bleeding, rather than glucocorticoid-induced mucosal injury, may be the predominant mechanism of upper gastrointestinal hemorrhage in this population.

*Helicobacter pylori* infection also increases UGIB risk (20–22). A Thai prospective case–control study found that *H. pylori* significantly associated with increased UGIB incidence in inpatients (23). A Spanish case–control study identified *H. pylori* as an independent UGIB risk factor in continuous low-dose aspirin users (24). However, this association was not consistently observed in certain populations. A cohort study on warfarin/direct oral anticoagulants (DOAC) users undergoing *H. pylori* eradication reported no significant UGIB risk difference between the eradication and *H. pylori*-negative groups in new warfarin starters (25). Another study in post-cardiac surgery ICU patients on stress ulcer prophylaxis found no link between *H. pylori* and UGIB (26). In our study, although UGIB patients had a higher *H. pylori* positivity rate than non-UGIB patients (28.1% vs. 17.8%), the difference was not significant, and a positive C13 urea breath test showed no significant association with UGIB on univariate Cox analysis.

The main causes of UGIB in patients with AILD undergoing long-term GC therapy can be roughly divided into two categories: esophageal and gastric varices caused by portal hypertension in cirrhosis or non-variceal bleeding, such as peptic ulcer (27). PPIs, as conventional acid-suppressing agents, primarily work by inhibiting H^+^-K^+^-ATPase activity to suppress gastric acid secretion and protect the mucosal barrier (28). They are mainly used for peptic ulcer bleeding caused by gastric acid erosion or drug-induced gastrointestinal bleeding. A systematic review and meta-analysis found that PPI, when used for > 1 month, can decrease the re-bleeding rate after endoscopic therapy in patients with cirrhosis for prophylaxis or emergency treatment purposes (29). It is also associated with lower short-term rebleeding and mortality in patients receiving esophageal variceal band ligation (30, 31). Another meta-analysis that re-evaluated the role of PPIs after endoscopic band ligation of esophageal varices (EBL) in patients with cirrhosis concluded that current evidence is insufficient to support routine PPI use after EBL (32). In our study, PPIs did not show a significant preventive effect against UGIB in patients with AILD treated with GC regardless of when PPIs were started (at baseline, within 1 year, or beyond 1 year) and regardless of how long they were used. A real-life experience aimed at evaluating the appropriateness of PPI prescriptions in hospitalized patients with cirrhosis reported that 47.4% of the inpatients and 34.7% at discharge had no valid indication for PPI administration. The most common inappropriate indication was portal hypertensive gastropathy, followed by treatment with GC and anticoagulants alone (33). This contrasts sharply with their established role in preventing drug-related bleeding, such as that caused by NSAIDs or antiplatelet agents (9, 10, 34–37).

Given our finding that routine PPI prophylaxis may not effectively reduce UGIB in patients with AILD on GC, alternative strategies merit consideration, including non-selective beta-blockers for variceal prophylaxis, selective monitoring of high-risk patients (defined by low platelets, significant varices, or PBC), correction of coagulopathy in cholestatic patients, endoscopic variceal ligation, and targeted PPI use in those with the peptic ulcer history or concomitant NSAID use.

This is the first study to investigate UGIB risk in patients with AILD receiving glucocorticoid therapy and to systematically evaluate the preventive role of PPIs. Our analysis integrates detailed clinical and laboratory data, including endoscopy findings and comprehensive liver function parameters, enabling more precise risk stratification. Our findings provide strong evidence that prophylactic PPI use offers no significant benefit in this population. However, several limitations should be acknowledged, including the retrospective single-center design, possible immortal time bias due to non-standardized PPI timing, limited statistical power from few events (*n* = 32), and possible UGIB misclassification without universal endoscopic confirmation. Furthermore, indication bias is a major potential confounder in this study. The proportions of patients with a history of peptic ulcer and those with C13 positivity were significantly higher in the PPI group than in the Non-PPI group. This indicates that PPI prescription was preferentially targeted at patients with preexisting gastrointestinal conditions. These reasons may affect the preventive effect of PPI on glucocorticoid-related UGIB.

Future large-scale, prospective, multicenter cohort studies are needed to validate our findings. Randomized controlled trials (RCTs) incorporating endoscopic evaluation could enable more precise bleeding risk stratification (e.g., via risk prediction models) to assess PPI benefit across different risk subgroups. Additionally, further research should explore the impact of PPIs on disease progression and long-term outcomes in patients with AILD to optimize overall management strategies.

## 5 Conclusion

In this retrospective cohort of 364 patients with AILD who received GC therapy, the incidence of UGIB was 8.8% over a median follow-up of 29 months. Lower platelet count and moderate to severe esophagogastric varices were independent factors for UGIB in patients with AILD receiving GC treatment. In this cohort, PPI users had a higher risk of bleeding; however, this association may be driven by confounding factors (such as more severe liver disease or comorbidities). Moreover, the addition of PPI at different time points or varying durations of PPI therapy did not significantly affect the risk of UGIB in this population. These findings suggest that the risk of UGIB is primarily attributed to portal hypertension rather than mucosal damage caused by GC. This implies that the primary strategy for preventing UGIB in patients with AILD undergoing GC should be to treat the underlying disease etiology, and to screen and manage esophagogastric varices, rather than to rely on routine acid suppression therapy with PPIs.

## Data Availability

The raw data supporting the conclusions of this article will be made available by the authors, without undue reservation.
