# Supplementary material for: Should proton pump inhibitors be used for glucocorticoid-related upper gastrointestinal bleeding prevention in patients with autoimmune liver disease? A real-world cohort study
Source: Front Immunol. 2026 Sep 10;17:1878948. doi: 10.3389/fimmu.2026.1878948 (PMC13601323; doi:10.3389/fimmu.2026.1878948)
Supplement: Supplementary file 1 [file Table1.docx]

**Supplement Table 1 Baseline Characteristics of Patients Non-PPI group and PPI group**

| Parameter | Total  n=364 | Non-PPI group  n=248 | PPI group  n=116 | P-value |
| --- | --- | --- | --- | --- |
| **Demographic** |  |  |  |  |
| Female(n,%) | 324(89.0) | 223(89.9) | 101(87.1) | 0.418 |
| Age (year) | 53(47-59) | 53(47-59) | 53(46-58) | 0.707 |
| FU Time(Mo) | 29(9-57) | 23(9-53) | 40(12-69) | 0.012 |
| **Diagnosis**(n,%) |  |  |  | 0.856 |
| AIH | 120(33.0) | 81(32.7) | 39(33.6) |  |
| PBC+AIH | 244(67.0) | 167(67.3) | 77(66.4) |  |
| **Laboratory** |  |  |  |  |
| PLT(10*9/L) | 115(75-179) | 119(74-181) | 107(79-179) | 0.944 |
| ALT (IU/L) | 60(32-109) | 59(32-109) | 62(31-109) | 0.842 |
| ALP(IU/L) | 158(108-254) | 157(111-248) | 161(103-300) | 0.548 |
| TBIL(μmol/L) | 23.90(14.22-48.67) | 23.45(14.25-44.37) | 25.00(14.15-52.92) | 0.758 |
| ALB(g/L) | 36.1±5.2 | 35.9±5.1 | 36.5±5.4 | 0.384 |
| APTT(s) | 38.45(33.10-43.27) | 38.25(32.82-42.67) | 38.95(33.50-43.67) | 0.502 |
| INR | 1.07(0.98-1.23) | 1.07(0.98-1.23) | 1.07(0.96-1.22) | 0.618 |
| IgG(g/L) | 21.20(17.12-25.80) | 21.45(17.62-26.60) | 21.00(16.95-24.87) | 0.350 |
| IgM(g/L) | 2.39(1.41-4.20) | 2.42(1.38-4.04) | 2.36(1.43-4.38) | 0.535 |
| **Complications** |  |  |  |  |
| C13-positivity(n,%) | 68(18.7) | 6(2.4) | 62(53.4) | <0.001 |
| Diabetes(n,%) | 19(5.2) | 13(5.2) | 6(5.2) | 0.978 |
| Hypertension(n,%) | 38(10.4) | 24(9.7) | 14(12.1) | 0.487 |
| Peptic Ulcer(n,%) | 18(4.9) | 5(2.0) | 13(11.2) | <0.001 |
| MS EGV(n,%) | 84(23.1) | 54(21.8) | 30(25.9) | 0.388 |
| **Medication** |  |  |  |  |
| Initial GC Dose(mg) | 40(30-50) | 40(30-40) | 40(30-40) | 0.711 |
| Duration of GC (Mo) | 10(4-25) | 10(4-22) | 12(4-30) | 0.231 |

*FU Time,* follow-up time*; Mo,* Month*; PLT*, platelet count; *ALT*, alanine aminotransferase; *ALP*, alkaline phosphatase; *TBIL*, total bilirubin; *ALB*, albumin; *APTT*, activated partial thromboplastin time; *INR*, International Normalized Ratio; *IgG*, immunoglobulin G; *IgM*, immunoglobulin M; *C13*, Carbon-13 urea breath test; *MS EGV*, moderate to severe esophagogastric varices; *GC*, glucocorticoid; *PPI*, proton pump inhibitors.

P values were between No-PPI group and PPI group

**Supplement Table 2 Baseline Characteristics of Patients according Duration of PPI Use**

| Parameter | Total  n=116 | Duration of use ≤14days  n=79 | Duration of use  >14 days  n=37 | P-value |
| --- | --- | --- | --- | --- |
| **Demographic** |  |  |  |  |
| Female(n,%) | 101(87.1) | 67(84.8) | 34(91.9) | 0.446 |
| Age (year) | 54(46-59) | 53(45-59) | 54(47-60) | 0.585 |
| FU Time(Mo) | 34(9-59) | 36(8-62) | 29(9-55) | 0.507 |
| **Diagnosis**(n,%) |  |  |  | 0.133 |
| AIH | 39(33.6) | 23(29.1) | 16(43.2) |  |
| PBC+AIH | 77(66.4) | 56(70.9) | 21(56.8) |  |
| **Laboratory** |  |  |  |  |
| PLT(10*9/L) | 111(76-172) | 108(74-180) | 124(80-148) | 0.689 |
| ALT (IU/L) | 48(25-94) | 39(22-82) | 57(32-109) | 0.103 |
| ALP(IU/L) | 154(96-226) | 152(100-215) | 156(93-237) | 0.974 |
| APTT(s) | 37.95±8.52 | 37.82±8.51 | 38.22±8.65 | 0.815 |
| IgG(g/L) | 19.38±7.27 | 18.94±6.95 | 20.31±7.94 | 0.350 |
| IgM(g/L) | 2.32(1.30-4.03) | 2.38(1.49-4.04) | 2.04(1.09-3.87) | 0.278 |
| **Complications** |  |  |  |  |
| C13-positivity(n,%) | 62(53.4) | 53(67.1) | 9(24.3) | <0.001 |
| Peptic Ulcer(n,%) | 13(11.2) | 6(7.6) | 7(18.9) | 0.137 |
| MS EGV(n,%) | 29(25.0) | 16(20.3) | 13(35.1) | 0.084 |

*FU Time,* follow-up time*; Mo,* Month*; PLT*, platelet count; *ALT*, alanine aminotransferase; *ALP*, alkaline phosphatase; *TBIL*, total bilirubin; *ALB*, albumin; *APTT*, activated partial thromboplastin time; *IgG*, immunoglobulin G; *IgM*, immunoglobulin M; *C13*, Carbon-13 urea breath test; *MS EGV*, moderate to severe esophagogastric varices.

P values were between Duration of use ≤14days and Duration of use >14 days

**Supplement Table 3 Comparison of survival curves and hazard ratio analysis**

|  |  | HR | 95%CI |
| --- | --- | --- | --- |
| PPI Use | Non-PPI group | Ref | - |
|  | PPI group | 2.631 | 1.281-5.405 |
| PPI treatment duration | Non-PPI group | Ref | - |
|  | PPI group | 1.212 | 0.438-3.356 |
| PPI Initiation Timepoint |  |  |  |
| Baseline | Non-PPI group | Ref | - |
|  | PPI group | 1.539 | 0.587-4.028 |
| Within 1 year after baseline | Non-PPI group | Ref | - |
|  | PPI group | 3.686 | 0.633-21.470 |
| Beyond 1 year after baseline | Non-PPI group | Ref | - |
|  | PPI group | 3.228 | 0.548-18.990 |

**Supplement Table 4 Sensitive analysis for the primary outcome (PPI Use and PPI treatment duration)**

|  | Methods | Group | HR | 95%CI | Log-rank  (P value) |
| --- | --- | --- | --- | --- | --- |
| PPI Use | Before adjust | Non-PPI group | Ref | - | - |
|  |  | PPI group | 2.631 | 1.281-5.405 | 0.008 |
|  | After adjust | Non-PPI group | Ref | - | - |
|  |  | PPI group | 1.311 | 0.839-2.047 | 0.234 |
| PPI treatment duration | Before adjust | Non-PPI group | Ref | - | - |
|  |  | PPI group | 1.212 | 0.438-3.356 | 0.426 |
|  | After adjust | Non-PPI group | Ref | - | - |
|  |  | PPI group | 2.039 | 0.976-4.256 | 0.057 |

Note: “adjust” means that we assumed all censored patients had experienced the primary outcome event.

**Supplement Table 5 Sensitive analysis for the primary outcome (PPI Initiation Timepoint)**

|  | Methods | Group | HR | 95%CI | Log-rank  (P value) |
| --- | --- | --- | --- | --- | --- |
| PPI Initiation Timepoint |  |  |  |  |  |
| Baseline | Before PSM | Non-PPI group | Ref | - | - |
|  |  | PPI group | 2.002 | 1.126-3.558 | 0.018 |
|  | After PSM | Non-PPI group | Ref | - | - |
|  |  | PPI group | 1.539 | 0.587-4.028 | 0.343 |
| Within 1 year after baseline | Before PSM | Non-PPI group | Ref | - | - |
|  |  | PPI group | 1.147 | 0.464-2.829 | 0.766 |
|  | After PSM | Non-PPI group | Ref | - | - |
|  |  | PPI group | 3.686 | 0.633-21.470 | 0.132 |
| Beyond 1 year after baseline | Before PSM | Non-PPI group | Ref | - | - |
|  |  | PPI group | 1.197 | 0.337-4.237 | 0.780 |
|  | After PSM | Non-PPI group | Ref | - | - |
|  |  | PPI group | 3.228 | 0.548-18.990 | 0.195 |

*PSM*, propensity score matching

**Supplement Table 6 Sensitive analysis for the primary outcome (PPI Initiation Timepoint)**

|  | Methods | Group | HR | 95%CI | Log-rank  (P value) |
| --- | --- | --- | --- | --- | --- |
| PPI Initiation Timepoint |  |  |  |  |  |
| Baseline | PSM | Non-PPI group | Ref | - | - |
|  |  | PPI group | 1.539 | 0.587-4.028 | 0.343 |
|  | PSM ( PU + C13 ) | Non-PPI group | Ref | - | - |
|  |  | PPI group | 2.684 | 0.741-9.721 | 0.132 |
| Within 1 year after baseline | PSM | Non-PPI group | Ref | - | - |
|  |  | PPI group | 3.686 | 0.633-21.470 | 0.132 |
|  | PSM ( PU + C13 ) | Non-PPI group | Ref | - | - |
|  |  | PPI group | 1.120 | 0.245-5.120 | 0.883 |
| Beyond 1 year after baseline | PSM | Non-PPI group | Ref | - | - |
|  |  | PPI group | 3.228 | 0.548-18.990 | 0.195 |
|  | PSM ( PU + C13 ) | Non-PPI group | Ref | - | - |
|  |  | PPI group | 6.050 | 0.117-312.4 | 0.371 |

*PSM*, propensity score matching; *PU*, peptic ulcer; *C13*, Carbon-13 urea breath test.

Note: “PSM ( PU + C13 )” means that peptic ulcer and Carbon-13 urea breath test status were included as covariates in the propensity score matching.
